# New insights in the clinical and translational relevance of miR483-5p in adrenocortical cancer

**DOI:** 10.18632/oncotarget.19118

**Published:** 2017-07-10

**Authors:** Francesca Salvianti, Letizia Canu, Giada Poli, Roberta Armignacco, Cristian Scatena, Giulia Cantini, Alessandra Di Franco, Stefania Gelmini, Tonino Ercolino, Mario Pazzagli, Gabriella Nesi, Massimo Mannelli, Pamela Pinzani, Michaela Luconi

**Affiliations:** ^1^ Department of Experimental and Clinical Biomedical Sciences “Mario Serio”, University of Florence, Florence, Italy; ^2^ Department of Translational Research and New Technologies in Medicine and Surgery, University of Pisa, Pisa, Italy; ^3^ Department of Surgery and Translational Medicine, University of Florence, Florence, Italy

**Keywords:** adrenal carcinoma, miRNA, liquid biopsy, rare tumor, quantitative real time RT-PCR

## Abstract

Adrenocortical cancer (ACC) is a rare aggressive malignancy. Recent ACC integrated genomics analysis contributed to redefine the risk groups on molecular basis, including tumor microRNAs (miRs), detectable also in the bloodstream. We developed a quantitative real-time (RT) assay for the measurement of miR483 and miR483-5p absolute levels in plasma samples. miR483/miR483-5p levels were evaluated in plasma samples of 27 patients with ACC before surgery and at follow-up.

Statistically significant differences in miR483-5p and miR483 levels were found between stage 1/2 and stage 3/4 ACCs in pre-surgery and post-surgery samples. ROC curve analysis of miR483–5p levels gave a prediction of the clinical stage (accuracy 0.917±0.084), with the best cut-off value of 0.221 ng/ml, prognosticating overall and recurrence-free survival. In a multivariate Cox analysis (HR 16.2, 95%CI[1.39-188.6, P<0.026]), miR483-5p was the only variable that significantly predicted recurrence, but not overall survival. In addition, miR483 and miR483-5p levels correlated with the number of circulating tumor cells (CTCs) detected in the same blood samples, independently of the timing of sampling. In conclusion, we demonstrated that miR483-5p absolute plasma levels in ACC patients are powerful molecular markers that may help in the follow-up of patients after surgery and chemotherapy, and contribute to more accurately classify and predict tumor progression.

## INTRODUCTION

Adrenocortical carcinoma (ACC) is a rare but often aggressive tumor, with dismal prognosis and limited therapeutic options. A complete resection of the mass is the mainstay treatment associated with adjuvant mitotane therapy at advanced stages. Although mitotane mechanisms of action are still poorly understood, its use has been proven to significantly increase disease-free survival [1].

Over the last years, the diagnostic and prognostic relevance of the pan-genomics profile clustering of tumors has been demonstrated for adrenocortical cancer [2, 3]. In addition to the profiles derived from the analysis of the characteristics of the primary tumor mass, information provided by the liquid biopsy components, i.e. miRNA (miR), free circulating DNA, and circulating tumor cells (CTCs), can better characterize tumors at a molecular level [4]. The liquid biopsy is a non-invasive test on blood samples, and provides biological material to monitor easily the tumor progression and response to therapies. Integration of this molecular information enables the identification of subgroups of malignant tumors with distinct molecular alterations and clinical outcomes, enabling the possibility of future personalized therapeutic approaches [5].

miRs are small non-coding RNA molecules that modulate expression of target genes post-transcriptionally by degrading mRNA or inhibiting translation [6]. Different miR profiles have been characterized in ACC [7, 8]. In particular, the two mature sequences of miR483, miR483-5p and miR483-3p, mapping in the second intron of the insulin-like growth factor 2 (IGF2) gene, have proved to be overexpressed both in the primary tumor [9-11] and the bloodstream [12-14], with a diagnostic and prognostic value [12-14]. Despite the significant association between high relative circulating levels of miR483 in ACC patients before surgery and shorter recurrence-free and overall survival, no attempt has been made so far to evaluate miR483 absolute circulating levels and their modulation during the post-surgery follow-up.

We measured miR483 and miR483-5p absolute plasma levels of a cohort of 27 ACC patients before and after surgery, uncovering correlations with disease stage and predictive values particularly for the -5p variant.

## RESULTS

### miR483 and miR483-5p detection in cell-free plasma samples

**Figure 1:**
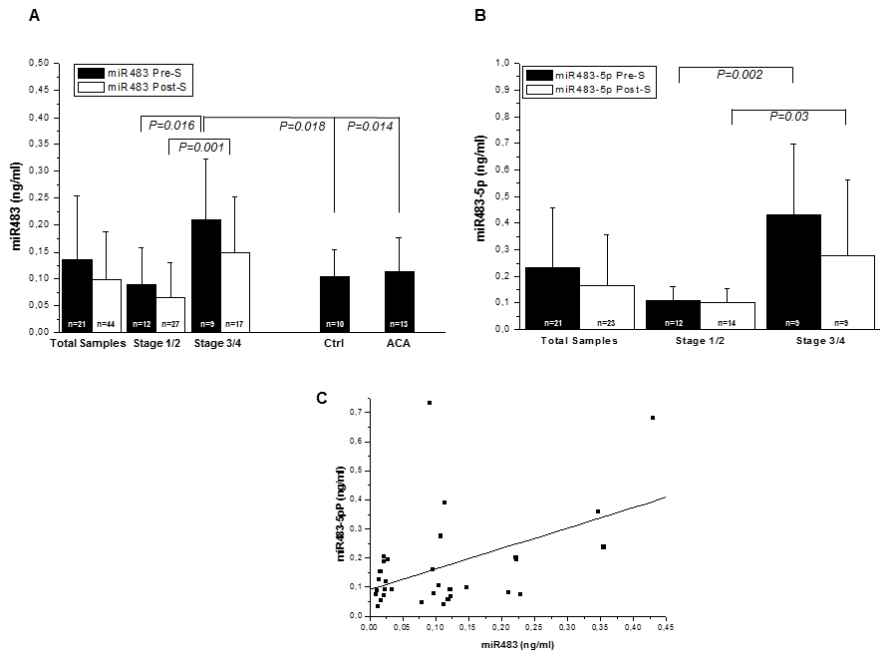
miR483 and miR483-5p detection in plasma samples miR483 **(A)** and mir483-5p **(B)** levels were measured in plasma samples obtained from n=27 patients with ACC, (pre surgery and post surgery), n=10 with ACA (pre-surgery) and n=10 healthy subjects. ACC samples were further stratified into low (st 1/2) and high (st 3/4) clinical stages, according to ENS@T classification [18]. Data is expressed as mean±SD of miR evaluated in the blood samples, with a follow-up median[95%CI] time from surgery of 13[4-27.5] months. The number of samples analyzed in each group is indicated (n). Statistics were performed with Student‘s t test for independent samples and P values are shown. **(C)** Positive correlation between miR483 and miR483-5p levels in samples from ACC patients. miR483-5p or miR483 were measured in n=32 pre-surgery and post-surgery samples;R=0.470, P=0.006.

Plasma samples were collected in pre-surgery (pre-S) and post-surgery (post-S) from a cohort of 27 patients affected by ACC, whose clinical characteristics are reported in Table 1. Mean follow-up was 17.6±17.3 months. Data was compared with that obtained by plasma analysis of 13 ACA patients, whose characteristics are shown in Table 2, and 10 healthy controls. miR483 absolute concentration was measured in all samples by means of a calibration curve, and expressed as ng/ml of plasma. miR483 absolute expression mean values in pre-S and post-S from ACCs, stratified into low (stages 1-2, St 1/2) and high (stages 3-4, St 3/4) pathologic stages, are displayed in Figure 1A, together with miR483 values from ACA and healthy control samples. A significant difference was found between the two stage groups in both miR483 values of pre-S (0.089±0.079 ng/ml and 0.210±0.113 ng/ml, St 1/2 and St 3/4, P=0.016, respectively) and post-S (0.066±0.0640 ng/ml and 0.150±0.102 ng/ml, St 1/2 and St 3/4, P=0.001, respectively) samples. The decrease observed in miR483 levels between pre-S and post-S samples within each stage group did not reach a statistical significance, probably due to the high variability among patients and the prevalence of sampling time very close to the operation, when miRs derived from the mass before being removed may be still present in the bloodstream. A significant difference in miR483 levels was seen between pre-S values of St 3/4 ACC and those of healthy controls [miR483 (ng/ml): 0.105±0.049, P=0.018] or ACAs [miR483 (ng/ml): 0.114±0.062, P=0.014]. There was difference between pre-S samples of St 1/2 ACCs and healthy controls or ACAs.

**Table 1:** Characteristics of ACC patients

	Mean (SD)	N Patients	%
**Age at surgery (years)**	45.3(16.2)	27	100
**Sex**			
**Male**		11	41
**Female**		18	59
**Secretion**		17	63
**Cortisol**		10	37
**Androgens**		8	30
**DHEAS**		2	7
**Progestins**		1	4
**Tumor Diameter (cm)**	8.8(4.9)	27	100
**Ki67 (%)**	26.8(24.7)	25	92.6
**WEISS**	6.5(1.9)	24	88.9
**Stage**			
**1**		6	22
**2**		12	44
**3**		4	15
**4**		5	19
**Metastases**	Lung, liver, bone, pancreas, contralateral adrenal	5	18.5
**Surgery**		27	100
**Surgery Resection**	**R0**	18	67
	**R2**	5	22
	**nd**	3	11
**MTT therapy**		21	78
**Other Chemotherapies (EDP, taxol)**		10	37
**Radiotherapy**		0	0
**Follow-up from surgery (months)**	17.6(17.3)	27	100
**Survival**		21	78

Etoposide-Doxorubicin-Cisplatin combined chemotherapy (EDP), Mitotane (MTT), not described (nd). Mean (SD) values for the indicated parameters are reported, along with the number (N) of patients and their percentage.

**Table 2:** Characteristics of ACA patients

	Mean (SD)	N Patients	%
**Age at diagnosis (years)**	57.4(15.2)	13	100
**Sex**			
**Male**		5	38
**Female**		8	67
**Secretion**			
**Cortisol**		6	46
**Non sec**		7	54
**Tumor Diameter (cm)**	3.6(1.2)	13	100
**Follow-up surgery/diagnosis (months)**	30.7(16.8)	13	100
**Surgery**		7	54
**Survival**		13	100

Mean (SD) values for the indicated parameters are reported, along with the number (N) of patients and their percentage.

We also assessed the absolute levels of the mature miR483-5p form using specific primers in samples screened for the total miR483 expression. We confirmed the presence of a significant difference between the two stage groups in both pre-S [miR483-5p (ng/ml):0.110±0.053 and 0.364±0.234, St 1/2 and St 3/4, P=0.002] and post-S [miR483-5p (ng/ml): 0.100±0.053 and 0.290±0.270, St 1/2 and St 3/4, P=0.03] levels, Figure 1B. Similarly to miR483, the decrease observed in miR483-5p levels between pre-S and post-S samples was not statistically significant albeit stage stratification, Figure 1B. A significant positive correlation was identified between total miR483 and miR483-5p levels, Figure 1C.

### Association of miR483-5p and miR483 levels with clinical parameters in ACC patients

When ACC clinical parameters were considered, a positive correlation was found between tumor stage and pre-S miR483-5p and miR483 levels (R=0.567, P=0.022, n=16, and R=0.527, P=0.036, n=16, respectively). Tumor diameter was only associated with pre-S miR483 levels (R=0.627, P=0.005, n=18,). There was no correlation with either Ki67% or Weiss score, even when stratifying ACC into two groups according to Ki67% value and Weiss score (cut-off of 10% for Ki67% and 6 for the Weiss score).

### Diagnostic and prognostic power of miR483-5p absolute plasmatic levels

**Figure 2:**
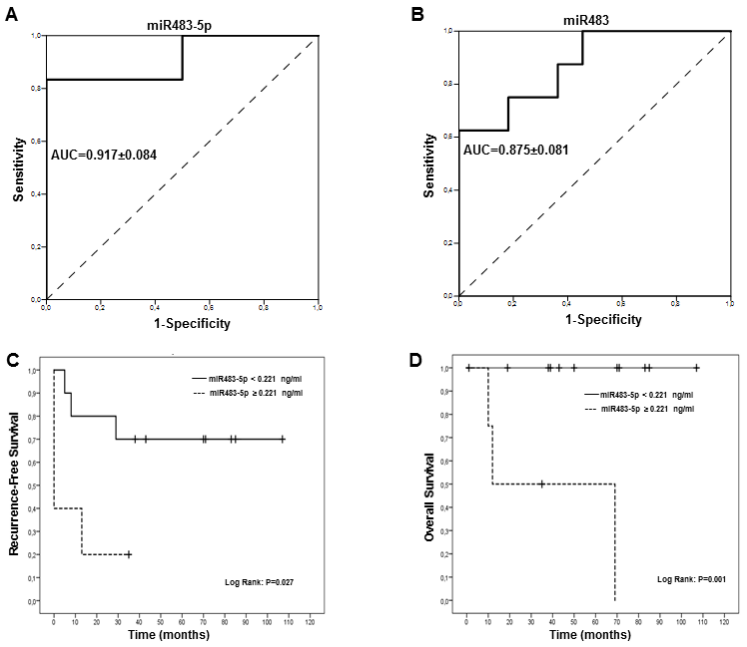
Receiver operator characteristics (ROC) and Kaplan-Meier (KM) analysis for miR483 and miR483-5p absolute levels in ACC patients ROC curve for miR483-5p **(A)** and miR483 **(B)** levels to discriminate between low (St1/2) and high (St3/4) clinical stages in ACC are shown. The ROC curve represents the relation between specificity and sensitivity for different thresholds miR (continuous line) compared to the diagonal reference line (dotted line). The area under the curve (AUC) represents the accuracy of the analysis, indicated with its SD. Kaplan–Meier survival analysis for circulating miR483-5p levels: **(**C**)** Recurrence free survival: miR483-5p levels ≥ 0.221 ng/ml cut off value derived from the ROC analysis are associated with a significantly lower recurrence free survival (Log Rank, P=0.027). **(**D**)** Overall survival: miR483-5p levels ≥ 0.221 ng/ml cut off value derived from the ROC analysis are associated with a significantly lower overall survival (Log Rank, P=0.001) P values were determined using a Log-Rank test.

ROC curve analysis was applied to assess the diagnostic accuracy of miR483 to distinguish between the low and high ACC stages. Better accuracy was achieved when considering pre-S miR483-5p levels (AUC of 0.917±0.084, p=0.007) than miR483 (AUC 0.875±0.081, p=0.006), Figure 2A and 2B. The best calculated cut-off values were 0.221 ng/ml, for miR483-5p (sensitivity=83.3% and specificity=100%), and 0.101 ng/ml, for miR483 (sensitivity=87.5% and specificity=63.6%).

To assess the prognostic ability of miR483-5p and miR483 to predict recurrence-free and overall survival, we constructed Kaplan-Meier curves stratifying patients into two groups on the basis of the cut-off values determined by ROC analysis (0.101 ng/ml for miR483 and 0.221 ng/ml for miR483-5p). Of the two parameters, only miR483-5p was able to prognosticate recurrence-free (Figure 2C) and overall survival (Figure 2D).

To adjust for imbalances in the distribution of potential prognostic factors between comparisons of recurrence-free survival and overall survival, univariate and Cox models were fitted to the data. Age and sex were included as covariates together with the miR483-5p cut-off level of 0.221 ng/ml. Tumor stage was not considered a covariate as it correlated with miR483-5p. On univariate analysis, only miR483-5p was associated with recurrence-free survival (P=0.049) but not with overall survival (P=0.412) (Table 3). After adjustments for age and sex, the patients with circulating levels of miR483-5p ≥ 0.221 ng/ml, maintained a significantly higher risk of recurrence (HR, 16.20; 95% confidence interval [CI], 1.39 to 188.6, P=0.026), Table 3.

**Table 3:** mi483-5p levels is a predictive factors for the risk of recurrence/metastasis in ACC, Predictive factors for recurrence/metastasis are reported according to Univariate and Multivariate Cox Analyses

Variable	Univariate			Multivariate		
	HR	95%CI	P	HR	95%CI	P
**miR483-5p**	4.67	0.99-21.88	*0.049*	16.2	1.39-188.6	*0.026*
**cut-off: 0.221 ng/ml**						
**Age**	1.03	0.97-1.10	0.277	1.02	0.96-1.08	0.522
**Sex**	1.03	0.26-4.11	0.971	0.65	0.98-4.23	0.654
**Stage**	2.43	0.97-6.08	0.58	-	-	-

The model for the multivariate analysis includes age (continuous variable) and sex (dichotomized variable with male as the reference category), in addition to miR483-5p category (dichotomized variable with miR483-5p < 0.221 ng/ml as the reference category); stage has not been included as it correlates with miR483-5p levels. HR: hazard ratio; 95%CI: 95% confidence interval. Significant P values are indicated in italics.

### Association between some parameters detected by the “liquid biopsy”: miR483-5p levels and circulating tumor cells

**Figure 3:**
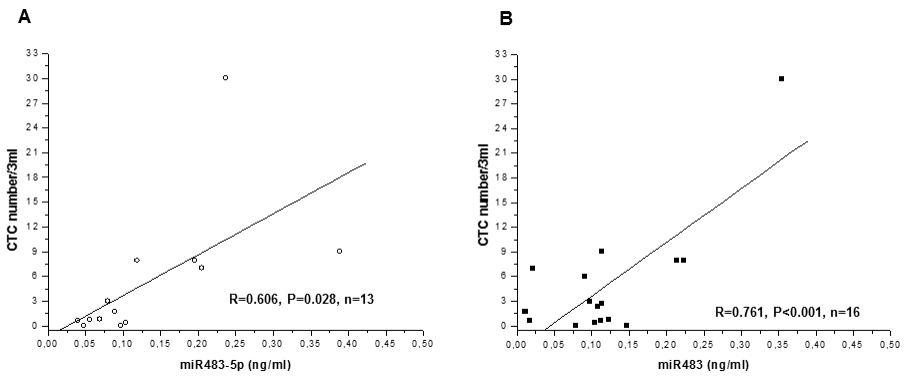
Correlations between circulating levels of miR483 or miR483-5p, circulating tumor cells (CTC) and mitotane levels in ACC patients CTC number positively correlates with miR483-5p **(A)** and miR483 **(B)** levels, as evaluated on the same samples from ACC patients (n=13), independently of the time of sampling.

In a small group of patients (n=13), CTCs were isolated and counted in the same pre-S (n=9) and post-S (n=7) blood samples in which miR483 and miR483-5p levels had been measured. A significant positive correlation was found between CTCs and both miR483-5p (Figure 3A) and miR483 (Figure 3B).

## DISCUSSION

The liquid biopsy is rapidly emerging as a powerful technique in oncology, not only for its diagnostic and prognostic uses, but also for monitoring the tumor progression through minimally invasive blood sampling. Interestingly, the liquid biopsy can provide important information on CTCs detached from the tumor mass, as well as on the molecular markers released by the tumor, such as circulating miR and free DNA.

miRs can be detected in blood samples of ACC patients paralleling the expression of the primary tumor [7, 8]. Among those miRs differentially expressed in malignant and benign adrenal cortical tumor tissues, [2, 3, 8-10, 12, 15], miR483 and its mature variant miR483-5p, are detected at higher levels in blood from patients with ACC compared to ACA [12, 14]. Moreover, increased levels of miR483-5p and decreased levels of miR195, showed prognostic value in a cohort of 21 ACCs stratified into non-aggressive and aggressive cancers [12]. However, the sensitivity of the miR quantification technique used was low, as miR483-5p in serum samples of non-aggressive tumors was under the detection limit of the method and no cut-off value derived from ROC curves and used for recurrence or survival analysis was given. In addition, miR483 quantification was normalized with spiked-in RNA methodology using exogenously added Cel-miR-39 [12]. It is still under debate as to which is the most accurate method for miR normalization in qRT-PCR analysis, as no reliable and stable endogenous housekeeping miR has been defined so far [14, 16]. To overcome problems regarding normalization of miR plasma levels, which may affect the validity of diagnostic and prognostic tests based on circulating miR measurement, we developed an absolute quantification method for miRs. This method is based on qRT-PCR using a standard curve, and the amount of miRNA is quantified by capillary electrophoresis. The absolute quantification strategy adopted renders the assay independent from the reference genes and makes it suitable for measuring the specific target miR in repeated samples during disease monitoring. Moreover, the used reference material derived from a human ACC (H295R) cell line is expected to more closely resemble miR characteristics than artificial miRs used for spiking in previous studies [12, 17]. Using this method, miR483 and miR483-5p plasma levels were significantly different in high-risk (stages 3 and 4, according to ENS@T, 18) and low-risk ACCs (stages 1 and 2), or ACAs and normal adrenal. On ROC curve analysis, miR483-5p showed better accuracy (AUC) than miR483, with the best cut-off value of 0.221 ng/ml differentiating between the two ACC groups. This cut off-value represents a sensitivity of 83% and a specificity of 100% of the method. Due to the absolute quantification of miR483 in ACC plasma samples based on miR483 measurement obtained in H295R positive control, this novel method of miR483/miR483-5p detection is reproducible and not affected by the above-mentioned analytical problems. Interestingly, despite the positive correlation found between total miR483 and miR483-5p, the latter variant displayed a better discriminant power between low- and high-stage ACC groups. In a Cox multivariate regression analysis, miR483-5p remained the best prognostic variable for recurrence risk. Notably, miR483 maps at 11p15.5 within the second intron of the insulin-like growth factor 2 (IGF2) gene [7], which is one of the most hyper-expressed genes in ACC [7, 8, 19], and is involved, although apparently not being sufficient in promoting ACC in the mouse [20, 21], in the multistep progression process underlying the carcinoma development [22]. miR483-5p is one of the mature variants derived from the processing of miR483. It has been demonstrated to self-enhance miR483 transcription [23] and hyper-activate IGF2 expression, by directly binding the P2 promoter of the IGF2 gene and decreasing its maternally-imprinted methylation [24]. Thus, miR483-5p represents one of the best markers for maintaining the self-fueling oncogenic activation of adrenocortical cancer cells.

Among the clinicopathologic parameters of ACC, only the tumor stage and diameter showed a positive correlation with miR483-5p. Likewise, we previously found a significant correlation of these two parameters with CTCs [23], one of the tumor markers obtainable from the liquid biopsy. CTC number and miR483-5p levels detected in the same blood samples of ACC patients showed a significant positive correlation. These findings validate not only the specific release of miR483 from the tumor mass (correlation with the diameter), but also the strict relationship between the corpuscular and the acellular part of the liquid biopsy.

Although our study confirms the reduction of miR483 circulating levels previously observed after surgery [12], this decrease is not significant in our series, probably due to the high variability among subjects. Moreover, miR levels may remain high in the bloodstream at short times of follow up from surgery, due to the high stability of miRs in circulation even for long period. Indeed, nearly 40% of the post-surgery samples in our study has been drawn in 6 months from surgery, when probably miRs derived from the tumor mass before surgery are still present.

Conversely, the statistically significant difference in miR483 and miR483-5p levels, detected between low and high stage groups before surgery, is also maintained at follow-up, suggesting that these miRs could be valuable markers of the tumor stage even in the long term. Moreover, the reduction observed after surgery and the correlation with the tumor diameter, further confirm specific release of miR from the tumor mass.

The main limitation of our study is the small cohort of ACC patients analyzed. However, ACC is a rare cancer, and our cohort of patients is well characterized according to the clinical data, and to date is the largest series analyzed for circulating miR483 and miR483-5p.

In conclusion, our study contributes to shed new light on the clinical diagnostic and prognostic power of miR483-5p detected in the bloodstream of patients with ACC. Furthermore, the quantitative method of absolute measurement of miR483-5p could be easily transferred to the routine analysis.

## PATIENTS AND METHODS

### Patients

The study included 40 patients affected by malignant (n=27 ACC) and benign (n=13 ACA) adrenocortical tumors, enrolled and evaluated at our University Hospital in Florence (AOU Careggi).

Plasma samples from healthy volunteers (n=10) were used as a control cohort of non-tumoral subjects for miR483 assessment.

The study was approved by the Local Ethical Committee and written informed consent to participate was obtained from all patients.

### Blood sample collection

For each subject, blood was collected in EDTA tubes, and plasma for miR extraction was obtained by centrifugation. In some patients (n=13) an additional 3 ml of total blood was processed within 3 hours after collection for CTC analysis.

### Plasma separation

Plasma was separated from blood collected in EDTA tubes by two centrifugation steps of 10 minutes at 4°C: the first at 1,600g, the second at 16,000g. Plasma aliquots were stocked at -80°C.

### RNA extraction

Reference RNA was extracted from the human cell line H295R by the miRNeasy Mini kit (Qiagen, Germany) following the protocol for Purification of Total RNA, including Small RNAs, from Animal Cells. Total cell-free RNA was extracted from 200 µl of plasma by using the miRNeasy mini kit, following the protocol for Purification of Total RNA, Including Small RNAs, from serum or plasma. Elution volume was 50 µl.

### Quantification of miR483

Quantification of miR483 and miR483-5p in cell-free RNA was performed by real-time quantitative RT-PCR (qRT-PCR) using the Taqman Micro RNA Reverse Transcription kit (Life Technologies, USA) and the following Taqman Small RNA assays (Life Technologies, USA): hsa-miR483 and hsa-miR483-5p. RT and qPCR reactions were performed according to the manufacturer’s protocol on the instrument 7900HT (Life Technologies). Samples were quantified by using a reference curve expressed as the total amount of miRNA in the reference sample (total RNA from H295R cell line). The amount of miRNA in the reference sample was quantified by capillary electrophoresis using the kit Small RNA assay on the Agilent 2011 Bioanalyzer (Agilent Technologies, USA). The range of the standard curve was 0.00523-5.23-pg miRNA. The quantity of miR483 and miR483-5p was calculated by interpolating the Cq values of qPCR on the standard curve and expressed as total miRNA equivalents. The results were normalized for the volume of plasma and expressed as ng equivalents/ml plasma (abbreviated in text and figures as ng/ml).

### CTC analysis

CTC analysis was performed on pre-surgery (n=9) and post-surgery (n=7) samples obtained from 13 patients with ACC, as previously described [25]. Briefly, the method consists of three sequential steps consisting of isolation of CTCs from blood by filtration on ScreenCell^®^ Cyto filtration devices (ScreenCell, Paris, France), followed by identification through validated morphometric criteria [25-27] and cell count. Identification of adrenocortical cell origin was confirmed by immunocytochemistry using the polyclonal antibody against the adrenocortical marker steroidogenic factor 1 (SF-1, cat #07-618 Upstate, Millipore, Billerica, MA).

### Histology and immunohistochemistry of the primary tumor

Histologic diagnosis was made by the referring pathologist on tumor tissue removed at surgery (ACC, n=27 and ACA, n=7). In 6 non-operated patients affected by non-functioning adrenal incidentaloma, the diagnosis of ACA was established based on the tumor characteristics by CT/MRI and unchanged imaging characteristics at least one year after diagnosis.

Tumor specimens were evaluated according to the Weiss system, which combines nine morphologic parameters: three related to tumor structure (description of cytoplasm, diffuse architecture and necrosis), three to cytology (atypia, atypical mitotic figures and mitotic count), and three to invasion (veins, sinusoids and tumor capsule). The presence of three or more criteria strongly correlates with malignant behavior [28].

Immunohistochemistry was performed on formalin-fixed and paraffin-embedded tissues using antibodies directed against adrenocortical markers, such as SF-1, MART-1, inhibin-alpha and synaptophysin, to define the adrenocortical origin of the tumor [25]. Ki67 index was evaluated as a proliferation marker to assess ACC prognosis [29]. Immunohistochemical analysis with mouse anti-human Ki67 monoclonal MIB1 antibody (Dako) was carried out utilizing the Ventana Benchmark XT system (Ventana Medical Systems). Nuclei were hematoxylin-counterstained, Ki67 positive nuclei were counted on 1,000 tumor cells and Ki67 was expressed as the percentage of proliferating cells. Negative controls were achieved by omitting the primary antibody.

Tumor stage was assessed according to the revised TNM classification of ACC proposed by the European Network for the Study of Adrenal Tumors (ENS@T) [18].

### Statistical analysis

All data was expressed as mean±SD. Statistical analysis was performed by SPSS 24.0 (Statistical Package for the Social Sciences, Chicago, USA) for Windows. The Kolmogorov–Smirnov’s test was used to verify the normal distribution of data, which was expressed as mean ± SD. Differences in continuous variables were analyzed by means of Student’s t test for independent data to compare two classes of data. Univariate correlations were carried out using Pearson’s test. Receiver operating characteristic (ROC) curve analysis was performed to calculate AUC and select the miR483 and miR483-5p values showing the best sensitivity and specificity for discriminating between low (St 1/2) and high (St 3/4) disease stages. Survival (overall and recurrence-free) curves were computed according to the Kaplan–Meier analysis and were compared by means of the log-rank test. A Cox proportional-hazards regression analysis was used in univariate and multivariate analyses to assess the predictive role of miR483 or miR483-5p cut-off values on disease recurrence and overall survival. P<0.05 value was considered statistically significant.

## Abbreviations

ACC: adrenocortical carcinoma
CTCs: circulating tumor cells
IGF2: insulin-like growth factor 2
miR: micro RNA
ROC: Receiver operator characteristics

## References

[1] Terzolo M, Angeli A, Fassnacht M, Daffara F, Tauchmanova L, Conton PA, Rossetto R, Buci L, Sperone P, Grossrubatscher E, Reimondo G, Bollito E, Papotti M (2007). Adjuvant mitotane treatment for adrenocortical carcinoma. N Engl J Med.

[2] Assié G, Letouzé E, Fassnacht M, Jouinot A, Luscap W, Barreau O, Omeiri H, Rodriguez S, Perlemoine K, René-Corail F, Elarouci N, Sbiera S, Kroiss M (2014). Integrated genomic characterization of adrenocortical carcinoma. Nat Genet.

[3] Zheng S, Cherniack AD, Dewal N, Moffitt RA, Danilova L, Murray BA, Lerario AM, Else T, Knijnenburg TA, Ciriello G, Kim S, Assie G (2016). Comprehensive pan-genomic characterization of adrenocortical carcinoma. Cancer Cell.

[4] Alix-Panabières C, Pantel K (2016). Clinical applications of circulating tumor cells and circulating tumor DNA as liquid biopsy. Cancer Discov.

[5] Faillot S, Assie G (2016). Endocrine tumours: the genomics of adrenocortical tumors. Eur J Endocrinol.

[6] Gregory RI, Shiekhattar R (2005). MicroRNA biogenesis and cancer. Cancer Res.

[7] Cherradi N (2016). microRNAs as Potential Biomarkers in Adrenocortical Cancer: Progress and Challenges. Front Endocrinol (Lausanne).

[8] Hassan N, Zhao JT, Sidhu SB (2016 Dec 20). The role of microRNAs in the pathophysiology of adrenal tumors. Mol Cell Endocrinol.

[9] Soon PS, Tacon LJ, Gill AJ, Bambach CP, Sywak MS, Campbell PR, Yeh MW, Wong SG, Clifton-Bligh RJ, Robinson BG, Sidhu SB (2009). miR-195 and miR-483-5p Identified as predictors of poor prognosis in adrenocortical cancer. Clin Cancer Res.

[10] Patterson EE, Holloway AK, Weng J, Fojo T, Kebebew E (2011). MicroRNA profiling of adrenocortical tumors reveals miR-483 as a marker of malignancy. Cancer.

[11] Duregon E, Rapa I, Votta A, Giorcelli J, Daffara F, Terzolo M, Scagliotti GV, Volante M, Papotti M (2014). MicroRNA expression patterns in adrenocortical carcinoma variants and clinical pathologic correlations. Hum Pathol.

[12] Chabre O, Libé R, Assie G, Barreau O, Bertherat J, Bertagna X, Feige JJ, Cherradi N (2013). Serum miR-483-5p and miR-195 are predictive of recurrence risk in adrenocortical cancer patients. Endocr Relat Cancer.

[13] Patel D, Boufraqech M, Jain M, Zhang L, He M, Gesuwan K, Gulati N, Nilubol N, Fojo T, Kebebew E (2013). MiR-34a and miR-483-5p are candidate serum biomarkers for adrenocortical tumors. Surgery.

[14] Szabó DR, Luconi M, Szabó PM, Tóth M, Szücs N, Horányi J, Nagy Z, Mannelli M, Patócs A, Rácz K, Igaz P (2014). Analysis of circulating microRNAs in adrenocortical tumors. Lab Invest.

[15] Özata DM, Caramuta S, Velázquez-Fernández D, Akçakaya P, Xie H, Höög A, Zedenius J, Bäckdahl M, Larsson C, Lui WO (2011). The role of microRNA deregulation in the pathogenesis of adrenocortical carcinoma. Endocr Relat Cancer.

[16] Mitchell PS, Parkin RK, Kroh EM, Fritz BR, Wyman SK, Pogosova-Agadjanyan EL, Peterson A, Noteboom J, O’Briant KC, Allen A, Lin DW, Urban N, Drescher CW (2008). Circulating microRNAs as stable blood-based markers for cancer detection. Proc Natl Acad Sci U S A.

[17] Roberts TC, Coenen-Stass AM, Wood MJ (2014). Assessment of RT-qPCR normalization strategies for accurate quantification of extracellular microRNAs in murine serum. PLoS One.

[18] Fassnacht M, Johanssen S, Quinkler M, Bucsky P, Willenberg HS, Beuschlein F, Terzolo M, Mueller HH, Hahner S, Allolio B, German Adrenocortical Carcinoma Registry Group, European Network for the Study of Adrenal Tumors (2009). Limited prognostic value of the 2004 International Union Against Cancer staging classification for adrenocortical carcinoma: proposal for a Revised TNM Classification. Cancer.

[19] Giordano TJ, Thomas DG, Kuick R, Lizyness M, Misek DE, Smith AL, Sanders D, Aljundi RT, Gauger PG, Thompson NW, Taylor JM, Hanash SM (2003). Distinct transcriptional profiles of adrenocortical tumors uncovered by DNA microarray analysis. Am J Pathol.

[20] Drelon C, Berthon A, Ragazzon B, Tissier F, Bandiera R, Sahut-Barnola I, de Joussineau C, Batisse-Lignier M, Lefrançois-Martinez AM, Bertherat J, Martinez A, Val P (2012). Analysis of the role of Igf2 in adrenal tumour development in transgenic mouse models. PLoS One.

[21] Heaton JH, Wood MA, Kim AC, Lima LO, Barlaskar FM, Almeida MQ, Fragoso MC, Kuick R, Lerario AM, Simon DP, Soares IC, Starnes E, Thomas DG (2012). Progression to adrenocortical tumorigenesis in mice and humans through insulin-like growth factor 2 and β-catenin. Am J Pathol.

[22] Gicquel C, Bertagna X, Gaston V, Coste J, Louvel A, Baudin E, Bertherat J, Chapuis Y, Duclos JM, Schlumberger M, Plouin PF, Luton JP, Le Bouc Y (2001). Molecular markers and long-term recurrences in a large cohort of patients with sporadic adrenocortical tumors. Cancer Res.

[23] Emmerling VV, Fischer S, Kleemann M, Handrick R, Kochanek S, Otte K (2016). miR-483 is a self-regulating microRNA and can activate its own expression via USF1 in HeLa cells. Int J Biochem Cell Biol.

[24] Zhang Y, Hu JF, Wang H, Cui J, Gao S, Hoffman AR, Li W (2017). CRISPR Cas9-guided chromatin immunoprecipitation identifies miR483 as an epigenetic modulator of IGF2 imprinting in tumors. Oncotarget.

[25] Pinzani P, Scatena C, Salvianti F, Corsini E, Canu L, Poli G, Paglierani M, Piccini V, Pazzagli M, Nesi G, Mannelli M, Luconi M (2013). Detection of circulating tumor cells in patients with adrenocortical carcinoma: a monocentric preliminary study. J Clin Endocrinol Metab.

[26] De Giorgi V, Pinzani P, Salvianti F, Panelos J, Paglierani M, Janowska A, Grazzini M, Wechsler J, Orlando C, Santucci M, Lotti T, Pazzagli M, Massi D (2010). Application of a filtration- and isolation-by-size technique for the detection of circulating tumor cells in cutaneous melanoma. J Invest Dermatol.

[27] Mascalchi M, Falchini M, Maddau C, Salvianti F, Nistri M, Bertelli E, Sali L, Zuccherelli S, Vella A, Matucci M, Voltolini L, Pegna AL, Luconi M (2016). Prevalence and number of circulating tumour cells and microemboli at diagnosis of advanced NSCLC. J Cancer Res Clin Oncol.

[28] Lau SK, Weiss LM (2009). The Weiss system for evaluating adrenocortical neoplasms: 25 years later. Hum Pathol.

[29] Morimoto R, Satoh F, Murakami O, Suzuki T, Abe T, Tanemoto M, Abe M, Uruno A, Ishidoya S, Arai Y, Takahashi K, Sasano H, Ito S (2008). Immunohistochemistry of a proliferation marker Ki67/MIB1 in adrenocortical carcinomas: Ki67/MIB1 labeling index is a predictor for recurrence of adrenocortical carcinomas. Endocr J.

